# The COSMOS Registry of CytoSorb Hemoadsorption Therapy in Critically Ill Patients: Protocol for an International, Prospective Registry

**DOI:** 10.2196/55880

**Published:** 2024-11-05

**Authors:** Fabio Silvio Taccone, Frank Martin Brunkhorst, Gabriella Bottari, Jorge Hidalgo, Andreas Kribben, Jean-Louis Teboul, Dana Tomescu, Teresa Klaus, Joerg Scheier, Efthymios Deliargyris, Ricard Ferrer

**Affiliations:** 1 Department of Intensive Care Hôpital Universitaire de Bruxelles (HUB), Université Libre de Bruxelles (ULB) Brussels Belgium; 2 Integriertes Forschungs- und Behandlungszentrum (IFB) Sepsis und Sepsisfolgen, Klinik für Anästhesiologie und Intensivmedizin Universitätsklinikum Jena Jena Germany; 3 Pediatric Intensive Care Unit Children Hospital Bambino Gesù, IRCCS Rome Italy; 4 General Intensive Care Unit and COVID-19 Unit Belize Healthcare Partners Belize City Belize; 5 Klinik für Nephrologie, University Duisburg-Essen University Hospital Essen Essen Germany; 6 Paris-Saclay Medical School, Paris-Saclay University, Le Kremlin-Bicêtre Paris France; 7 Department of Anesthesiology and Critical Care “Carol Davila Bucharest Romania; 8 CytoSorbents Europe GmbH Berlin Germany; 9 CytoSorbents Corporation and CytoSorbents Medical Inc. Princeton, NJ United States; 10 Intensive Care Department Vall d'Hebron University Hospital, Shock, Organ Dysfunction and Resuscitation Research Group (SODIR) Barcelona Spain

**Keywords:** extracorporeal blood purification, CytoSorb, hemoadsorption, hemoperfusion, adsorption, hyperinflammation, sepsis, septic shock, liver failure, rhabdomyolysis, hospital care, mortality

## Abstract

**Background:**

Extracorporeal blood purification with CytoSorb has been increasingly used as an adjunctive therapy in several hyperinflammatory critical care conditions, as well as to remove elevated levels of myoglobin or bilirubin in patients with rhabdomyolysis or liver failure. Despite the increasing worldwide use of hemoadsorption, data from large international multicenter studies are still lacking.

**Objective:**

The COSMOS (CytoSorb Treatment Of Critically Ill Patients) registry is a company-sponsored registry by CytoSorbents Corporation and CytoSorbents Medical Inc. and will provide a data repository and reporting infrastructure for the surveillance of CytoSorb use in real-world critical care settings in an unselected, critically ill patient population. The gathered data will serve as a comprehensive resource to assess the effects of such therapy on patients’ management.

**Methods:**

The international COSMOS registry is collecting prospective data for patients treated with CytoSorb during routine care in various critical care indications, based on the decision of the treating physicians. Data are collected at baseline, during CytoSorb therapy, 24 hours thereafter, at discharge from the intensive care unit and the hospital, and on day 90. Key outcomes assessed include change in inflammatory biomarkers, vasopressor requirements, fluid balance, organ function and organ support, length of intensive care unit and hospital stay, occurrence of adverse events, and mortality.

**Results:**

The COSMOS registry started with the inclusion of the first patient on July 15, 2022, and is now actively enrolling in 4 countries (Germany, Spain, Portugal, and Italy), with plans to expand to other countries outside of Europe. An initial readout is planned for presentation at an international Critical Care conference in 2024.

**Conclusions:**

The COSMOS registry is intended to provide comprehensive real-world data on patient outcomes with CytoSorb in various critical care indications, thereby contributing to optimization of patient selection, timing of initiation, and dosing of hemoadsorption treatment.

**Trial Registration:**

ClinicalTrials.gov NCT05146336; https://clinicaltrials.gov/study/NCT05146336

**International Registered Report Identifier (IRRID):**

DERR1-10.2196/55880

## Introduction

### Background and Rationale

Prolonged and excessive inflammation, characterized by the persistent release of several mediators into the blood stream in response to different stimuli, is believed to contribute to multiple organ failure, leading to a high risk of mortality [1,2]. Blood purification has been increasingly used for treating critically ill patients with excessive inflammation and can be considered as an umbrella for different procedures, subdivided into hemofiltration, hemoadsorption, and plasma-processing techniques, including plasmapheresis and plasma exchange [3-5].

The concept of hemoadsorption refers to the adhesion of circulating molecules such as cytokines to the surface of the respective sorbent material. Different hypotheses have been developed to explain possible beneficial effects of removing such mediators from the bloodstream, including lowering pro- and anti-inflammatory mediators below a threshold to limit organ damage [6] and restoration of a cytokine gradient between tissue- and blood-promoting leukocyte chemotaxis [7]. This pathophysiological rationale and the growing number of clinical studies have led to the hypothesis that reducing inflammatory mediators within the systemic circulation could restore immune homeostasis and positively affect patient outcomes. Currently, there are several extracorporeal technologies available that offer potential benefits in blood purification for managing life-threatening conditions. These include CytoSorb (CytoSorbents), oXiris (including the AN69-ST; Baxter), Jafron HA-series (including HA-230, HA-280, HA-330, HA-330-II, and HA-380; Jafron), Seraph 100 Microbind Affinity Blood Filter (Seraph100; ExThera Medical), and Polymyxin B hemoperfusion (PMX-HP; Toraymyxin, Toray Medical Co., Ltd.). These devices vary in their specific modes of action and target substances for removal, approved indications, and documented clinical experiences, including any potential impact on drug removal [8-10].

The CytoSorb device is a Conformité Européenne labeled whole blood hemoadsorption device. The device was registered in 2011, and the number of patients treated with CytoSorb has continued to grow.

A recent randomized controlled trial (RCT) in healthy volunteers provided definitive mechanistic evidence of CytoSorb’s ability to decrease systemic cytokine levels after intravenous administration of endotoxin in an established sepsis model [11]. These findings support the approved indications of CytoSorb therapy for the treatment of a variety of clinical conditions in which excessively high plasma levels of inflammatory mediators occur, such as septic or vasoplegic shock, acute respiratory distress syndrome (ARDS), and critical illness requiring extracorporeal membrane oxygenation (ECMO) therapy. Additional application fields for CytoSorb include use of the device for removal of elevated levels of bilirubin and myoglobin in patients with life-threatening conditions, such as liver failure [12] or rhabdomyolysis [13], as well as the intraoperative removal of ticagrelor and rivaroxaban during cardiothoracic operations with cardiopulmonary bypass [14].

Despite the increasing worldwide use of blood purification therapies, large international, multicenter studies and robust evidence of clinical outcome improvement are still lacking [15,16]. Consequently, questions about the appropriate application modalities, especially regarding timing and dose, remain unanswered. Also, some conflicting data showing neutral or even negative outcomes have been published, underlining the need for further conclusive evidence generation [17-19]. A recent systematic review showed no mortality benefit of the therapy in a very heterogenic patient group and underlined the need to systematically identify patients likely to respond to the therapy with clinical benefits in a reproducible manner [16].

**Figure 1:**
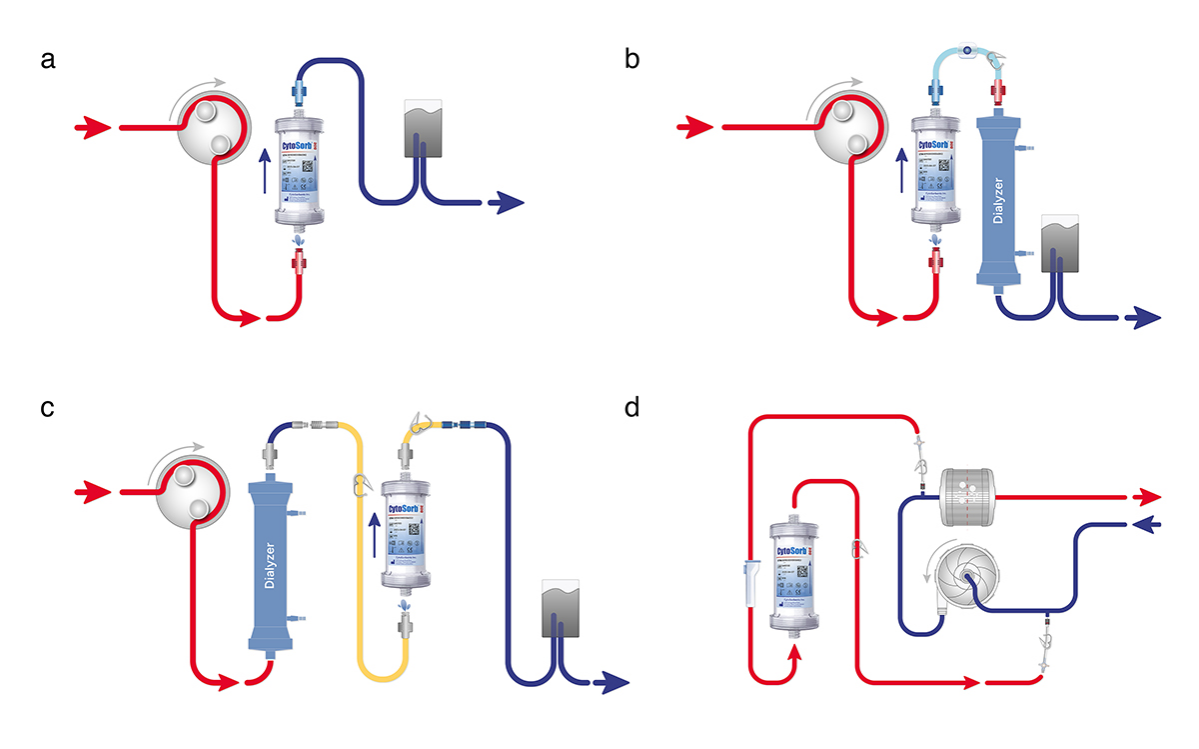
Setup integration of CytoSorb into different platforms: (A) hemoperfusion; (B) continuous renal replacement therapy (CRRT) predialyzer; (C) CRRT postdialyzer; and (D) extracorporeal membrane oxygenation with bypass.

The hemoadsorption device can be easily integrated into concomitant continuous renal replacement therapy or into a dedicated hemoperfusion circuit (Figure 1), as well as into a bypass within the ECMO circuit or a cardiopulmonary bypass circuit.

### Objectives of the COSMOS Registry

The aim of the COSMOS (CytoSorb Treatment Of Critically Ill Patients) registry is to capture real-world clinical use patterns and clinical outcomes associated with the utilization of the CytoSorb device and to help further refine best practices for the therapy. There is a well-known heterogeneity of treatment effects in critically ill patients [20], which is why collecting registry data is a valuable tool, making it easier to identify those patients who would potentially benefit most from the therapy. Also, treatments of critically ill children are included as a predefined subcohort, allowing the collection of multicenter data, thereby expanding the available evidence derived from single-center reports on limited patient numbers. Additionally, the registry will also serve as a crucial part of the postmarket clinical follow-up measures as required by Medical Device Regulation [21].

## Methods

### Study Setting

The COSMOS registry is an ongoing, observational, prospective, and international data collection platform for patients receiving CytoSorb devices during routine clinical care. Site selection was performed according to experience with the hemoadsorption therapy (total number of treatments per year), usual indications for use, estimated patient number contributions, experience in study conduct and electronic data collection, as well as personnel capacity and overall interest in participation based on a dedicated site qualification questionnaire. The treatment strategy and patient’s management are determined by the physician and not by the registry protocol. For all site personnel, including the principal investigator, subinvestigators, and clinical research coordinators, training on the latest hemoadsorption therapy recommendations is provided to ensure that the users are aware of the current understanding of best practice with CytoSorb and consider these aspects in their decision-making on therapy management [22,23]. Additionally, each investigator is encouraged to review the most recent CytoSorb instructions for use to assess contraindications, warnings, and precautions for treating potential patients. In order to best represent real-world practice and to avoid selection bias, sites are asked to include consecutive patients receiving the CytoSorb device. All clinical data from critically ill patients treated with the device after the date of approval of the institutional review board (IRB) or independent ethics committee (IEC) are considered “prospective data.”

The registry collects patient-level data with the use of the CytoSorb device in the following different clinical indications (Table 1).

The study may be discontinued at the discretion of the investigator (or designee) or sponsor if any of the criteria mentioned in Textbox 1 are met.

**Table 1:** Different indications for CytoSorb included in the COSMOS (CytoSorb Treatment Of Critically Ill Patients) registry.

Category	Clinical indication
Hyperinflammation	Septic shock Hemophagocytic lymphohistiocytosis CRS^a^ Chimeric antigen receptor therapy CRS
Cardio and pulmonary	Cardiogenic shock Acute respiratory distress syndrome Lung failure with venovenous extracorporeal membrane oxygenation and cardiac failure with extracorporeal life support system
Liver and kidney	Acute liver failure or acute-on-chronic liver failure Rhabdomyolysis
Infectious disease	COVID-19 Influenza Dengue
Others	Postoperative vasoplegic shock Burns Pancreatitis Overdose drug removal

^a^CRS: cytokine release syndrome.

**Study discontinuation criteria.**

- Medical or ethical concerns
- Safety issues pertaining to adverse device events (ADEs) or serious adverse events (SADEs) unknown to date
- Changes in accepted clinical practice making the continuation of a registry unwise
- Difficulties in patient recruitment
- Sponsor’s decision

### Eligibility Criteria

The registry population will be comprised of critically ill patients, including children who undergo treatment with the CytoSorb device as part of their routine clinical care. To best represent real-world practice and avoid selection bias, participating sites will ideally include all patients receiving the CytoSorb device consecutively, without preselection (Textbox 2).

**Inclusion and exclusion criteria.**

**Inclusion criteria**

- CytoSorb device utilization
- Informed consent for prospective registry participation

**Exclusion criteria**

- Use of CytoSorb device for antithrombotic drug removal only
- Intraoperative use of CytoSorb device during cardiac surgery only

### Ethical Considerations

This registry is being conducted in accordance with International Organization for Standardization (ISO) 14155:2020; the Declaration of Helsinki; the standards of Good Clinical Practice, as defined in the International Conference on Harmonization E6 Guideline for Good Clinical Practice; as well as all other local legal requirements. Prior to initiation of the registry, IRB and IEC approval must be received for the protocol, sample informed consent form (ICF), and any other documents that pertain to patient information and recruitment methods such as advertisements. All subsequent protocol amendments and changes to the ICF must also be submitted and approved by the IRB and IEC. The initial international ethics approval at Vall D’Hebron University Hospital, Barcelona, Spain, is PR(AG)86/2022. Each center must obtain approval from its responsible IRB before activation.

To collect in-hospital and postdischarge data, the investigator or designee must obtain a signed, IRB- and IEC-approved ICF from patients or their legally authorized patient representatives when the patient is identified as a potential candidate for the registry (refer to Multimedia Appendix 1 for the ICF). Consent is obtained either from the patient or, according to standard procedures in emergency situations, via the legally authorized patient representative. The ICF contains all the elements required by ISO14155:2020 and only includes data collection, as the indication of treatment with CytoSorb itself is solely at the discretion of the treating physician. In cases where patients are unable to give consent, a declaration of consent must be obtained from a legal representative, taking into account the patient’s presumed willingness. The legal representative may be an authorized representative appointed by a power of attorney or a court-appointed caregiver (health care task group) according to local regulations. For children, dedicated ICFs are provided. Patients who are not able to give their consent initially but who become able to during their stay in hospital will be informed about the registry when they are able again to consent, and then the written informed consent on participation in the registry is obtained. Any patient has the right to withdraw from the registry at any time without penalty or loss of benefit. There is no direct financial compensation for participants.

For each enrolled participant, an electronic case report form (eCRF) will be maintained. All clinical data will be extracted from medical records by the site investigator(s) and their designees. Data accuracy will be verified by centralized monitoring. Data will be entered in a deidentified manner; that is, patients who consented will be assigned a consecutive patient identification only traceable by the site. The eCRF will be available for inspection by the sponsor and its designees before, during, and upon completion of the registry. Patient rights regarding information, correction, deletion, and general handling of data will be respected according to the European General Data Protection Regulation. A full data readout is performed at least annually. The data are treated confidentially at all times and are protected against unauthorized access. In the event of premature termination of the registry, no further data will be collected. Transmission of registry-related data, for example, for statistical analysis, to countries with a level of data protection that may differ from that of the European Union, such as the United States, takes place exclusively in deidentified form. This ensures data confidentiality, as data cannot be assigned to a specific or identifiable actual person. Publication of the results of the registry study will be in anonymous form only.

### Data Capture and Outcomes

As per the registry protocol, there are no prespecified assessments or medical treatments to be performed other than a follow-up telephone call at 90 days (plus an additional 20 days if needed for administerial reasons) after the start of CytoSorb treatment. Data collection is performed based on medical chart review and transcription of deidentified data collected from hospital admission through discharge. The following key parameters are included (Textbox 3).

Data from the following time points will be collected if available (Textbox 4).

**Key parameters.**

- Demographics
- Baseline characteristics including Acute Physiology And Chronic Health Evaluation II score, respective Pediatric Index of Mortality-3 score for children, and Charlson Comorbidity Index score
- Indication for CytoSorb treatment
- Details on the extracorporeal circuit used (eg, type of platform, blood flow rate, and anticoagulation regimen), integration of CytoSorb, and duration of treatment
- Changes in Sequential Organ Failure Assessment scores or subscores and Pediatric Logistic Organ Dysfunction-2 score for children
- Changes in inflammatory biomarkers (C-reactive protein, procalcitonin, leukocytes, interleukin-6, and other interleukins)
- Laboratory results from hematology, chemistry, coagulation, and arterial blood gas analysis as well as additional drug level measurements
- Duration of organ support therapies (number of days on extracorporeal membrane oxygenation or extracorporeal life support system, number of days on continuous renal replacement therapy, number of days on mechanical ventilation, and number of days on concomitant procedures like therapeutic plasma exchange)
- Daily fluid balance
- Vasopressor requirements (type, dose, and duration)
- Ventilation parameters
- Liver disease assessment (eg, hepatic encephalopathy, bilirubin, bile acids, model for end-stage liver disease score, respective pediatric end-stage liver disease score for children, European Association for the Study of Liver—Chronic Liver Failure score, Maddrey score, and Lille score)
- Acute kidney injury assessment, including Kidney Disease Improving Global Outcomes severity score and myoglobin and creatine kinase levels for rhabdomyolysis
- Critical illness polyneuropathy
- Cumulative blood products
- Concomitant therapies including plasmapheresis and medication
- Length of intensive care unit (ICU) and hospital stay
- ICU and in-hospital mortality
- Safety performance of CytoSorb device, including collection of any unexpected serious adverse device effects
- Cases of device deficiency
- Long-term follow-up on day 90 (+20) including 90-day mortality and cause of death
- Study exit: vital status and registry exit date

**Data collected.**

- Baseline (at intensive care unit [ICU] admission)
- Before start of CytoSorb therapy (maximum 24 hours prior)
- During CytoSorb therapy
- After CytoSorb therapy (maximum 24 hours afterward)
- At discharge from ICU
- At discharge from hospital
- Follow-up on day 90 (+20)

A device deficiency is defined as any inadequacy of a medical device with respect to its identity, quality, durability, reliability, usability, safety, or performance, including malfunctions, use errors, and inadequate labeling [22]. Following the same standardization, a serious adverse device event (SADE) is defined as an adverse device event (ADE) that has resulted in any of the consequences characteristic for a SADE, with an unexpected SADE (USADE) being defined as a SADE that by its nature, incidence, severity, or outcome has not been identified in the current risk assessment. SADE and USADE will immediately be analyzed by the sponsor regarding possible root cause and possibility of reoccurrence. In addition, all statutory reporting duties will be fulfilled. In the case of USADE, the investigator has the same notification duties and reporting timelines as for SADE.

### Analysis and Statistical Methods

Descriptive statistics and patient data listings will be used to summarize the data. Continuous variables will be summarized by nonmissing values (n), mean and SD (in case of normal distribution), median, 25th and 75th percentiles (in case of nonnormal distribution), and minimum and maximum. Categorical variables will be summarized by frequencies and percentages. Stratification of descriptive categories within treatment groups may occur if such elaborations are thought to be useful. Inferential statistical tests may be used, as appropriate, with an α critical value of .05 to indicate statistical significance. Inferential statistical tests will be 2-sided, unless specified otherwise. Also, 95% CIs may be used to compare event rates to a historical and clinically meaningful standard or to investigate the course of biomarkers (or certain laboratory values) after CytoSorb therapy. Multivariate analysis may be used to determine independent predictors of study outcomes. Missing data is unavoidable for critically ill patients treated in the intensive care unit (ICU). The principle of analyses is to only include the nonmissing observations. For inflammatory biomarkers or laboratory values that are highly associated with the use of hemoadsorption devices, sensitivity analyses will be performed by applying a multiple imputation method under the missing-at-random assumption.

Unless specified otherwise, minimum and maximum will be displayed to the same level of precision as the observed value. Mean and median will be displayed to 1 level of precision greater than the observed value. The SD and SE will be displayed to 2 levels of precision greater than the observed value. All *P* values will be rounded to and displayed as 3 decimals. If a *P* value less than .001 occurs, it will be shown in tables as <.001.

## Results

The COSMOS registry is an international registry executed in countries where the device is approved and routinely used in everyday clinical practice. It started with the inclusion of the first patient on July 15, 2022. There is no prespecified sample size for the registry, but the current estimation is that with annual enrollment of approximately 300 patients, the registry can reach up to 3000 patients in 10 years. The COSMOS registry is now actively enrolling in 4 countries (Germany, Spain, Portugal, and Italy), with plans to expand to other countries outside of Europe. As of August 1, 2024, a total of 199 patients are included in the registry.

## Discussion

### Overview

The previous CytoSorb international registry collected patient data from May 2015 to December 2020 and was independently led as an investigator-initiated project by the Centre for Clinical Studies at the Jena University Hospital, Germany [24]. At its closure, 46 sites had included a total of 1434 patients showing improvements in cardiovascular and pulmonary Sequential Organ Failure Assessment scores and a reduction in procalcitonin, C-reactive protein, and interleukin-6 levels [24]. However, several important parameters were not captured, for example, lactate and hemodynamic data, due to a lack of knowledge of hemoadsorption treatment characteristics at the time of conceptualization of the project. Also, the definition of sepsis and septic shock changed in 2016 [25]. Furthermore, other indications for blood purification, including bilirubin and myoglobin removal, were not yet covered at the time by the Conformité Européenne label and were therefore not considered in the initial design of the Jena registry.

Existing literature in the field of CytoSorb treatment mainly consists of smaller studies, case series, and case reports [16], including studies that showed neutral or negative results [17-19]. In the following paragraphs, we summarize the current understanding of the pathophysiology and rationale behind the most common use of CytoSorb in critically ill patients. Of note, so far, evidence is still scarce with a lack of larger RCTs in the field. In general, pooled analysis of such heterogenous patient groups is difficult, especially given the methodological limitations of these studies [26].

### Septic and Vasoplegic Shock

The clinical conditions of sepsis and septic shock arise from a dysregulated host reaction to an infection, causing organ malfunction. The excessive release of inflammatory mediators beyond the local area in response to the infection characterizes the syndrome, leading to an overshooting inflammatory response from the host (known as a cytokine storm). However, comprehension of the fundamental pathophysiology behind organ and especially vascular dysfunction, including inadequate vascular response to vasoconstrictors (vasoplegia) caused by sepsis, remains incomplete. Existing data support the concept of sepsis-associated cytokine release as the driving force in refractory vasoplegic shock [27,28]. Both sepsis and septic shock represent 2 of the main causes of death in ICUs worldwide [29,30]. This considerable mortality is at least in part attributable to the inadequacy of current treatment options. The only causal therapeutic options are comprised of infectious source control and antibiotic therapy, while stabilization of the cardiac and respiratory systems is considered supportive measures. However, there are several reasons why CytoSorb therapy might be able to provide benefits for critically ill patients in the ICU who develop severe organ dysfunction due to a dysregulated immune response (ie, often termed as “hyperinflammation” and “cytokine storm”). Existing clinical data on the use of CytoSorb hemoadsorption in patients with sepsis support the concept that a reduction in inflammatory mediators, but certainly also of other, yet unknown substances, can result in improved hemodynamics, reduced needs for catecholamines, reversal of shock, and improved outcomes [31-33]. The effect of hemoadsorption therapy on mortality as a primary end point has not yet been evaluated in a prospective randomized manner, but retrospective studies suggest significant survival benefits in the CytoSorb-treated group compared to matched controls [34,35]. Next to sepsis, vasoplegic shock also occurs in the context of noninfectious triggers such as polytrauma, severe burns, severe acute pancreatitis, and cardiogenic shock [29,36]. Additionally, vasoplegic shock is commonly seen postoperatively after cardiac and other major surgeries [37,38]. Patients with postoperative vasoplegia will be included in this registry; however, intraoperative use of the device during cardiac surgery will not be included. The separate STAR (Safe and Timely Antithrombotic Removal) registry is dedicated for the indication of intraoperative antithrombotic removal with CytoSorb hemoadsorption (NCT04976530).

### ARDS and ECMO

ARDS is characterized by bilateral interstitial edema of the lungs, resulting from increased capillary leakage. If the condition advances to severe ARDS, one sole lifesaving option available is frequently ECMO. Nevertheless, this approach is expensive, burdensome, and exceedingly invasive, requiring specialized personnel, and is typically only available in specialized facilities. It is crucial to note that the application of ECMO itself may exacerbate the underlying hyperinflammatory state [39]. Recent reports also suggest that patients treated for severe ARDS who received blood purification treatment show improvements in oxygenation, as evidenced by an increased partial pressure of oxygen to fraction of inspired oxygen ratio [40-44].

### COVID-19

Based on the emerging knowledge that hyperinflammation may play a key role in respiratory failure in patients with COVID-19, CytoSorb therapy has been used in this setting with over 7500 patients treated worldwide (as of December 2021), including the United States under Food and Drug Administration–issued emergency use authorization [45-49]. The recently released results from the multicenter US CytoSorb therapy in COVID-19 registry showed high survival in the most critically ill patients requiring life support on ECMO [43,50]. The same rationale for using CytoSorb in COVID-19 also applies to other viral infectious etiologies leading to severe respiratory failure, such as dengue fever or influenza [51-55].

### Liver Failure

Currently, the medical world fundamentally distinguishes between acute liver failure (including hyperacute or fulminant) and acute-on-chronic liver failure, but all types of liver failure are associated with very high mortality irrespective of the presence of preexisting liver disease [56,57]. For most patients, the only definitive treatment is liver transplantation, but availability is limited. Several artificial liver support systems have been used with the aim of bridging by detoxification in patients with liver failure [56]. CytoSorb has been shown to be effective for the removal of bilirubin [12,58,59] and bile acids [60,61].

### Rhabdomyolysis

Additionally, hemoadsorption therapy has been increasingly used to remove myoglobin [13,62,63]. In cases of rhabdomyolysis, efficient removal of myoglobin is crucial to protect the patient from acute kidney injury as well as for organ recovery [64].

Furthermore, children will also be included in the registry, capturing additional specific parameters such as Pediatric Index of Mortality-3 and Pediatric Logistic Organ Dysfunction-2 scores. The instructions for use of the CytoSorb device do not specify patients’ age limitations but only mention that for treatment of patients weighing less than 45 kg, discretion should be used. The minimum treated body weight, reported in the literature, is 3.5 kg [65]. An ongoing multicentric observational study in Italy (CYTOPED) has already demonstrated safety and feasibility of the use of CytoSorb in the first 50 children and will allow further investigation into other aspects of the therapy in this patient cohort [66].

### Responsibilities and Dissemination

A scientific committee has been established to provide scientific oversight of the registry. This is led by the registry’s principal investigator and composed of internationally recognized investigators and experts in the different application fields of extracorporeal blood purification as well as the sponsor’s medical director of the trial. The scientific committee will be responsible for any changes to the registry design, the decisions for interim data analyses according to the statistical analysis plan, and the data readout strategy, including presentations at medical conferences and publications. Reports of the annual evaluations of the registry data and retrospective inspection of their own data will be made available to the participating registry centers. The scientific committee and the sponsor may appoint other independent committees to resolve specific problems such as verifying patient classification, adverse events adjudication, or publication policy. Operational execution of the trial will be performed by a contract research organization (EvidentIQ), with oversight and management performed by the sponsor’s clinical team.

### Strengths and Limitations

The COSMOS registry is a high-fidelity, systematic data collection platform for CytoSorb treatment across multiple critical care indications and highly representative of real-world practice [33,42,67]. Real-world evidence (RWE), as captured with registries, is particularly useful for expanding the evidence base to encompass populations of patients who are not well represented in RCTs but who may benefit from the intervention in question. RWE is also critical in the setting of complex, rapidly evolving treatments or overall rare indications, where RCT design cannot answer all the relevant questions or is not feasible. RWE is essential to better define a treatment’s safety profile under routine clinical conditions, particularly in the longer term. These uses enhance clinical knowledge and patient care with approved agents and devices. While RWE has many uses, it also has its limitations [68]. Efforts are being made to develop best practices for the mitigation of common biases in the design of real-world studies. These include ensuring that the data collected are comprehensive, accurate, and complete, by using validated data sources and conducting a thorough evaluation of the quality of data sources to assess their reliability and validity. Also, we aim to include a diverse study population to ensure that the results are generalizable to different patient groups. Engaging with all stakeholders to inform study design and ensure the research addresses relevant and meaningful questions is crucial for valid results of this registry.

### Conclusions

The intent of the COSMOS registry is to inform data-driven optimization of CytoSorb therapy across a broad variety of indications and to provide important safety information fulfilling regulatory requirements on postmarket surveillance. Furthermore, the current design of the COSMOS registry allows the potential for future registry-embedded RCTs [69] to answer specific prespecified hypotheses, a process that will require separate protocols, consent, and ethics approval.

## Appendix

Multimedia Appendix 1 Informed consent form.

## Abbreviations

ADE: adverse device event
ARDS: acute respiratory distress syndrome
COSMOS: CytoSorb Treatment Of Critically Ill Patients
ECMO: extracorporeal membrane oxygenation
eCRF: electronic case report form
ICF: informed consent form
ICU: intensive care unit
IEC: independent ethics committee
IRB: institutional review board
ISO: International Organization for Standardization
RCT: randomized controlled trial
RWE: real-world evidence
SADE: serious adverse device effect
STAR: Safe and Timely Antithrombotic Removal
USADE: unexpected serious adverse device effect

## Data Availability

Data sharing is not applicable to this paper as no datasets were generated or analyzed during this study. Data management plan, annotated electronic case report forms, and informed consent forms are available on request.
