# Supplementary material for: The COSMOS Registry of CytoSorb Hemoadsorption Therapy in Critically Ill Patients: Protocol for an International, Prospective Registry
Source: JMIR Res Protoc. 2024 Nov 5;13:e55880. doi: 10.2196/55880 (PMC11576605; doi:10.2196/55880)
Supplement: Multimedia Appendix 1 [file resprot_v13i1e55880_app1.pdf]

International registry on the use of CytoSorb® in the critical care setting –

the COSMOS Registry

Participant Information

Please read the following text carefully before making a decision. If there is anything you do not understand, please ask the study physician responsible for you.

Note to patient: You may not be able to decide for yourself whether or not to participate in this study. It is then customary to involve a legal representative who will be asked to decide whether participation in the study is in the best interest of that person, taking into account his or her likely wishes.

For the attention of the legal representative: Dear Madam / Dear Sir, the person you represent is not in a position, due to their clinical situation, to decide in full awareness of the implications of doing so, about participation in a clinical trial. You are therefore asked to decide whether he/she should participate in this clinical trial, taking into account his/her likely wishes. If the patient has flatly refused study participation when he/she was still capable of consent, this must of course be honored.

In the remainder of this document, the sentences are worded as if we are directly addressing the person you represent.

Dear Patient,

We would like to ask you whether you would like to participate in the above-mentioned clinical study. You will be informed about the study during a detailed medical consultation.

Your participation in this clinical study is voluntary. You can withdraw from the study at any time without giving reasons. Refusal to participate or early withdrawal from this study will not adversely affect your medical care.

Clinical trials are necessary to obtain reliable new medical research results. However, an indispensable prerequisite for conducting a clinical trial is that you give your written consent to participate in this clinical trial. Please read the following text carefully as a supplement to the information interview with your physician and do not hesitate to ask questions.

Please sign the consent form only

- if you fully understand the nature and procedure of the clinical study,
- if you are willing to consent to participate, and
- if you are aware of your rights as a participant in this clinical trial.

The responsible ethics committee has issued a favorable opinion on this clinical study, as well as on this patient information and consent form.

Aim of the study

The aim of this clinical study is to create a registry of data on the treatment of patients using the CytoSorb medical device. This is a type of database with information about patients with similar medical conditions and their treatments. This registry will be used to learn more about how CytoSorb can be used to reduce cytokine and/or bilirubin or myoglobin levels in the blood. In addition, the registry data will help to improve the safety profile of the medical device CytoSorb in this field of application.

Study procedure

This clinical trial is being conducted internationally at multiple sites, and a total of approximately 3,000 people will participate.

Participation in the registry study has no influence on treatment. This is the sole responsibility of the treating physician. By participating, you will not receive any additional examinations or therapies.

If you agree to participate in this study, researchers will collect your medical history and current and future CytoSorb medical treatment information that you receive as part of your intensive care treatment. This information will include, but not be limited to, your laboratory results,

medications, treatments and procedures.

Furthermore, you will be contacted by telephone 90 days after your CytoSorb treatment to record how you are doing by means of a few questions.

You will only have to spend a small amount of time as part of the registry study.

Risks

For this study, data will be collected as part of your standard medical care. There are no health risks associated with data collection. Your medical information will be handled in strict compliance with the European General Data Protection Regulation (GDPR). Furthermore, your data will be pseudonymized, i.e. the data can only be reassigned to you with the help of additional information, which will be stored separately and with restricted access. The process of pseudonymization is explained in more detail in the section "Data protection".

There are confidentiality risks (e.g., the possibility of identifying you) associated with any collection, storage, and transmission of data in the context of research projects. These risks cannot be completely eliminated and increase the more data can be linked together. See the "Data protection" section for a more detailed explanation of how your privacy is protected.

Benefit

You are not expected to get any direct health benefit from your participation in this clinical trial.

The investigators hope that this registry study can help improve CytoSorb therapy and its use in intensive care patients in order that future patients receive better and safer treatment.

Insurance

Because your doctor will make decisions about your treatment regardless of your registry study participation, it falls under the purview of your health insurance. Patients are not at higher risk by participating in this registry study compared to non-study patients. Therefore, no separate patient insurance is required for this registry study. There are no changes to your treatment as a result of registry study participation.

Legal requirements stipulate that approved medical devices that are on the market must be insured by the manufacturer within the framework of a so-called product liability obligation. This also applies outside of studies and applies to the CytoSorb medical device used in your standard treatment.

Privacy Policy

The information collected about you is called "data" or "study data" in this document. The study data will include information from past as well as future entries in your medical record during the time you are participating in the study.

Your treating physician and his or her staff, if any, will collect information about you that is relevant to this study. Specifically, this includes information about your age, gender, physical measurements (height and weight), ethnicity, relevant medical history information, physical examination data, vital signs, laboratory results, any medications you are taking, and complications (adverse events) and/or effects of the medical device. Furthermore, data from your intensive care and CytoSorb treatment will be used and analyzed for the registry study.

Access to the data, from which you are directly identifiable, will be given to the lead study physician and other study center staff involved in the clinical trial or your medical care. In addition, authorized and confidential representatives of the sponsor CytoSorbents Corporation as well as representatives of domestic and/or foreign health authorities and the respective competent ethics committees may have access to this data to the extent necessary or required to verify the proper conduct of the clinical study. All persons who have access to these data are subject to the applicable national data protection regulations and/or the EU General Data Protection Regulation (GDPR) when handling the data.

The data will be treated confidentially at all times. The data will be forwarded by your treating physician in a pseudonymized form to the sponsor of the study (CytoSorbents Corporation) or to agencies commissioned by him for the purpose of scientific evaluation.

Pseudonymized means that no information of names or initials will be used, but only a consecutive patient number ("pseudonymization" is the processing of personal data in such a way that the data can no longer be attributed to a specific data subject without the addition of further information, provided that this additional information is kept separately and is subject to technical and organizational measures to ensure that the data cannot be attributed to any data subject). A list is stored in the study center on which the patient names are assigned to the patient numbers of the registry study. This list is kept separately at the study center and is subject to technical and organizational measures to ensure that your personal data cannot be assigned by unauthorized persons. Only the study director and his or her expressly authorized deputies have access to this list.

Information entered into this registry study may be shared with other researchers and regulatory agencies. Data entered into the registry may be used to generate medical or scientific reports that may be viewed by the public. In either case, your identity will not be known.

In accordance with the European General Data Protection Regulation (EU-GDPR), you have the right to:

- Information about the processing of your data, and to receive a copy of the relevant personal data free of charge,
- Correction or deletion of your data,

- Restriction of processing (only storage is still possible),
- Objection to processing,
- Data transfer,
- Revocation of your given consent with effect for the future,
- Complaint to the data protection supervisory authority.

The *[Institution]* and CytoSorbents Corporation are responsible for data processing within this study. You have the right to obtain information about your personal data and to have it corrected in the event of an error. To do so, please contact the study center contact listed below.

The following data protection officers are also available to answer any questions you may have.

The contact details of the Institution's Data Protection Officer are as follows:

[Data Protection Officer of Institution]
[address]
[phone]
[e-mail]

The contact information for the Data Protection Officer appointed by the Sponsor, CytoSorbents Corporation, is as follows:

h3ko Vertriebsgesellschaft mbH
Pappelallee 64
16359 Biesenthal, Germany
phone: +49 (0)30 549 887 013
e-mail: dataprotection@cytosorbents.com

In addition, you have the right to lodge a complaint with the competent supervisory authority:

[Data Protection Officer of competent data protection supervisory authority]
[address]
[phone]
[e-mail]

Your consent forms the legal basis for the processing of your personal data. You can revoke your consent to the collection and processing of your data at any time without giving reasons. After your revocation, no further data will be collected about you. However, the data collected until revocation may continue to be processed within the scope of this clinical study.

The expected duration of the clinical trial is 10 years (until 2032). The data will be retained for 10 years after the study has ended or been discontinued. The data are secured against unauthorized access.

There are confidentiality risks associated with any collection, storage, use, and transmission of

data (e.g., the possibility of identifying the individual). These risks cannot be completely eliminated and increase the more data can be linked together. The sponsor of the study assures you that it will do everything possible according to the current best practice to protect your privacy and will only pass on data to bodies that can demonstrate an appropriate data protection concept. Medical risks are not associated with the data processing.

Your data may also be transferred to recipients in countries outside the EU if one of the following conditions is met:

- The European Commission has determined that the country has an adequate level of data protection under the law,

  or, if this has not been done:

- CytoSorbents Corporation / [*Institution*] agrees with research partners on contractual data protection clauses adopted or approved by the European Commission or the relevant supervisory authority. You may obtain a copy of these data protection clauses.

In addition, however, it may also be the case that data is to be transferred to research partners in third countries for which neither of these two conditions is met. These countries may have a lower level of data protection than the EU. CytoSorbents Corporation assures that it will contractually obligate the research partners to also comply with the EU data protection level as far as legally possible in these cases. Nevertheless, there is a risk that government or private entities may access your data even though this would not be permitted under European data protection law. In addition, you may have fewer or less enforceable data subject rights there and there may be no independent supervisory authority to assist you in exercising your rights.

This also applies to the United States of America (USA). Here, there is currently no decision by the European Commission that an adequate level of data protection exists there. With your consent, you agree that the data may also be transferred to this country. For this purpose, the data will be transferred exclusively in a pseudonymized form (see above), in order to protect your data as best as possible.

Voluntariness of participation

Your participation in this study is voluntary. You may revoke your consent to participate at any time without giving any reason and without any disadvantage to you. Please address your revocation to contact at the study center listed below.

Expense allowance

There is no compensation for participation in the registry study. However, you will not incur any costs as a result of your participation.

Contact details for questions

If you have any further questions in connection with this clinical study, your study doctor and his/her staff will be happy to answer them. They will also be happy to answer questions concerning your rights as a patient and participant in this clinical trial.

Contact details: *[e.g. study center, study physician]*

Can be reached at: phone: *[phone]*

e-mail: *[e-mail]*

International registry on the use of CytoSorb® in the critical care setting –

the COSMOS Registry

Declaration of Consent

I have been informed in writing and verbally about the above study and all my questions have been answered.

I am voluntarily participating in this study.

I know that I can withdraw my consent to participate at any time without giving reasons and without any disadvantages.

I know that no subject and/or commuting accident insurance has been taken out for this study.

I agree that data collected in the course of the study may be recorded on questionnaires and / or electronic data carriers and analyzed without mentioning my name (pseudonymized). I also agree that the study data may be used in an anonymized form for scientific presentations and publications.

My data may be passed on in a pseudonymized form as described in the information document. This includes disclosure to the USA for the purpose of scientific evaluation to CytoSorbents Corporation, 305 College Road East, Princeton NJ 08540, USA (sponsor of the study) or entities contracted by the sponsor.

I explicitly consent to the transfer of my pseudonymized data to the USA. Without this consent to the transfer of the pseudonymized data to the USA, participation in the registry is not possible.

I have received a copy of the information document and signed consent form.

Place, Date	*Name in block letters*	*Signature of Participant*

OR

Place, *Date*	*Name in block letters*	*Signature of Legal representative*

AND

Place, Date	*Name in block letters*	*Signature of consenting physician*
